# Validation of a portable video head impulse test using an iPod touch and an oral stabilization three-dimensional (3D) - printed mount: a method-comparison study

**DOI:** 10.3389/fneur.2026.1831063

**Published:** 2026-06-22

**Authors:** Masao Noda, Tatsuaki Kuroda, Akiko Umibe, Yumi Dobashi, Reiko Tsunoda, Hiroaki Fushiki

**Affiliations:** 1 Department of Otorhinolaryngology, Mejiro University Ear Institute Clinic, Saitama, Japan; 2 Department of Otolaryngology and Head and Neck Surgery, Jichi Medical University, Tochigi, Japan; 3 Kuroda Ear, Nose and Throat Clinic, Kumamoto, Japan; 4 Department of Otorhinolaryngology, Head and Neck Surgery, Dokkyo Medical University Saitama Medical Center, Saitama, Japan

**Keywords:** laterality, method comparison, neuro-otology, portable vestibular testing, smartphone-based testing, unilateral vestibular hypofunction, vestibulo-ocular reflex, video head impulse test

## Abstract

**Background:**

Although video head impulse testing (vHIT) is widely used to assess semicircular canal function, access to quantitative vestibular testing remains limited. Smartphone-based vHIT systems with goggle-based fixation have been described, but larger validation studies and improved fixation stability are needed. We evaluated a portable vHIT application using an iPod touch with an oral stabilization three-dimensional (3D)-printed mount to determine whether it detects laterality in unilateral vestibular hypofunction and is noninferior to a medical-grade device for a patient-level laterality endpoint.

**Methods:**

Thirteen patients with unilateral vestibular hypofunction and 14 healthy volunteers underwent testing with the portable and medical-grade vHIT systems on the same day. The primary endpoint was the patient-level laterality difference, defined as the difference between unaffected and affected vestibulo-ocular reflex (VOR) gain. Noninferiority was assessed using the two-sided 95% confidence interval (CI) for the mean between-device difference with a prespecified margin of 0.15. Agreement was evaluated using Bland–Altman analysis. Secondarily, absolute VOR gain agreement between devices was evaluated. False-positive classifications in healthy volunteers were assessed at the ear and subject levels using a gain threshold of <0.78.

**Results:**

Both systems showed higher VOR gains on the unaffected side (paired *t*-test: medical device, *p* = 2.44 × 10^−7^; portable vHIT, *p* = 1.02 × 10^−8^). Mean laterality difference was 0.518 ± 0.180 for the medical-grade device and 0.453 ± 0.118 for the portable system. The between-device difference was −0.065 (95% CI: −0.149 to 0.018), supporting noninferiority. Bland–Altman analysis demonstrated a bias of −0.065, with 95% limits of agreement from −0.337 to 0.207. For absolute VOR gain, average between-device bias was small, but individual-ear agreement remained variable. In healthy volunteers, abnormal classifications occurred in 0 of 28 ears and 0 of 14 subjects with the medical-grade device, and in 2 of 28 ears (7.1%) and 2 of 14 participants (14.3%) with the portable system.

**Conclusion:**

The portable vHIT system detected laterality in unilateral vestibular hypofunction and met noninferiority criteria relative to a medical-grade device. Larger studies are warranted to refine quality control and define individual-ear absolute VOR gain agreement.

## 1 Introduction

The video head impulse test (vHIT) is a widely used vestibular function test that quantifies vestibulo-ocular reflex (VOR) gain during brief, high-acceleration head impulses (1). Because it can be performed rapidly and provides side-specific information, vHIT has become an important tool in the clinical assessment of dizziness and imbalance, particularly in patients with suspected unilateral vestibular hypofunction (2, 3). Despite its clinical utility, broader adoption of quantitative vHIT systems may be limited by equipment costs, device availability, and operational constraints in routine practice. Expanding access to objective vestibular testing therefore remains an important clinical goal.

Portable digital approaches may help address these barriers by improving accessibility and simplifying deployment in outpatient and community settings (4–6). However, adapting vHIT to a portable platform presents several technical challenges. Reliable measurements depend on stable coupling between the recording device and the head, accurate tracking during rapid motion, and consistent acquisition across repeated impulses. Even minor instability during testing can affect gain measurements and increase variability, particularly in healthy individuals.

A prototype smartphone-based vHIT system using goggle-based fixation and representative waveform output has previously been reported (7). That work established feasibility but also highlighted the need for quantitative validation in larger cohorts and raised concerns regarding fixation stability and slippage associated with goggle-based mounting. The next step in the development of a portable vHIT platform therefore involves not only refinement of the fixation method but also direct comparison with a medical-grade reference system using clinically interpretable endpoints.

In the present study, we developed a portable vHIT application running on an iPod touch, combined with an oral stabilization three-dimensional (3D)–printed mount. Unlike goggle-based fixation, this approach is designed to improve coupling stability during head impulses by using an oral support structure to reduce relative motion between the device and the head. We then conducted a method-comparison study against a medical-grade vHIT system in patients with unilateral vestibular hypofunction and in healthy volunteers.

The aims of this study were twofold. First, we evaluated whether the portable vHIT system could discriminate between the affected and unaffected sides in patients with unilateral vestibular hypofunction. Second, we assessed whether the portable system was noninferior to a medical-grade vHIT system for a patient-level laterality endpoint, defined as the difference between unaffected and affected VOR gains. Agreement between systems was evaluated for this endpoint, and abnormal classifications in healthy volunteers were examined at both the ear and subject levels.

## 2 Materials and methods

### 2.1 Study design

This prospective method-comparison study was conducted to evaluate a portable vHIT system in comparison with a medical-grade vHIT system. Patients and healthy volunteers underwent testing with both systems during the same session on the same day.

### 2.2 Participants

Patients were consecutively recruited individuals who presented to the Mejiro University Ear Institute Clinic with symptoms of dizziness or vertigo and were diagnosed with unilateral vestibular hypofunction. The affected side was clinically determined prior to vHIT testing. Symptom duration was recorded categorically and spontaneous nystagmus findings were documented, as summarized in Table 1. Neurotological examinations and brain magnetic resonance imaging (MRI) and/or computed tomography (CT) were performed to exclude central vestibular disorders. Healthy volunteers were independently recruited, were not age-matched to the patient group, and had no history of vestibular or neurological disease.

**Table 1:** Clinical characteristics of patients with unilateral vestibular hypofunction (*n* = 13).

Case	Age	Sex	Disorder	Affected side	Symptom duration	Spontaneous nystagmus
1	58	M	Vestibular neuritis	Right	2	Leftward
2	79	M	Vestibular neuritis	Right	3	None
3	55	M	Ramsay Hunt syndrome	Right	3	None
4	76	M	Unknown cause	Right	3	None
5	82	F	Unknown cause	Right	3	Leftward
6	85	M	Ramsay Hunt syndrome	Left	3	Rightward
7	64	M	Vestibular neuritis	Right	3	None
8	54	F	Postoperative acoustic neuroma	Left	3	None
9	68	M	Unknown cause	Right	2	None
10	76	M	Vestibular neuritis	Left	3	None
11	55	F	Vestibular neuritis	Right	3	unknown
12	60	M	Vestibular neuritis	Left	3	Rightward
13	52	F	iSSNHL with vertigo	Right	1	Leftward

F, female; M, male; iSSNHL, idiopathic sudden sensorineural hearing loss. Symptom duration was categorized as follows: 1, 3–6 months; 2, 6–12 months; 3, >12 months.

### 2.3 Ethics statement

This study was approved by the Medical Research Ethics Committee of Mejiro University (approval number: Medical 24-021). Written informed consent was obtained from all participants prior to enrollment. All procedures were conducted in accordance with the principles of the Declaration of Helsinki.

### 2.4 Devices and portable vHIT system

Medical-grade vHIT was performed using the EyeSeeCam system (Interacoustics, Denmark), and analysis was conducted using the OtoAccess Database (version 2.1.0). The system provided VOR gain values for rightward and leftward head impulses and reported within-test variability metrics.

The portable vHIT system consisted of a dedicated iOS application (vHIT96da; version 6.7) running on a 7th generation iPod touch (iOS 15.8.4), together with a 3D–printed oral stabilization mount. The iPod touch was selected for this platform-specific implementation because of its low weight (88 g) and 120-frame/s camera capability, as described in our prototype report (7).

Eye movements in the portable system were analyzed using a corneal reflex–based method rather than infrared pupil tracking. Specifically, motion of the LED reflection point on the cornea was extracted from high-speed camera images using OpenCV-based image processing, while head movement was estimated using the built-in gyroscope. Gyroscope data were recorded at 10-ms intervals during each head impulse. The recording workflow included calibration and correction for an approximately 10-ms lag between the onset of video recording and gyroscope data acquisition, consistent with our prior prototype description (7).

For between-device comparability, the portable system was configured to calculate VOR gain at 60 ms after impulse onset, matching the gain definition used in the EyeSeeCam reference analysis. This study was not designed as a software engineering report, and lower-level iOS interface details were not prospectively archived; however, the operational measurement workflow and signal-processing approach are described here to enhance reproducibility.

**Figure 1:**
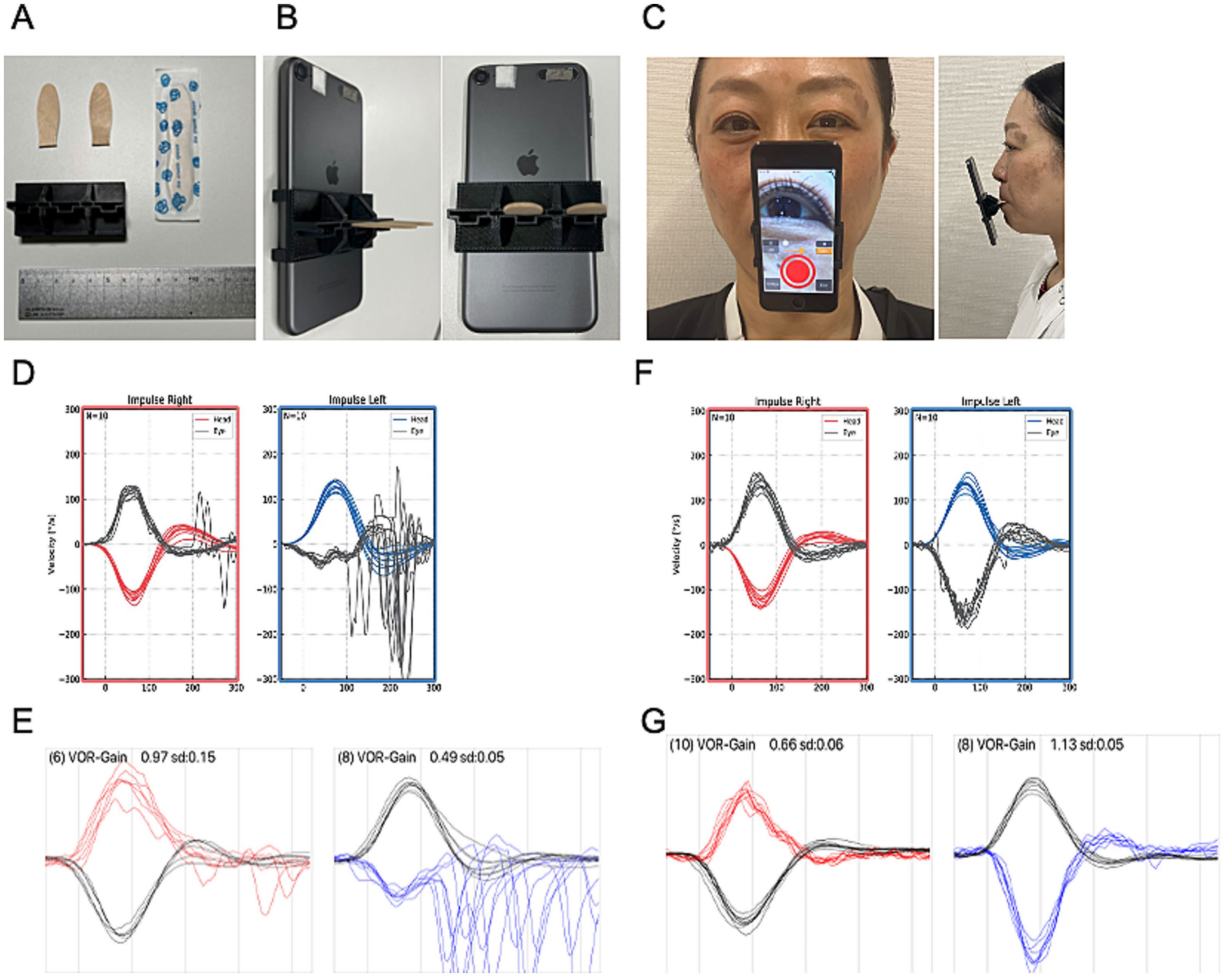
Portable vHIT system and representative recordings. **(A)** Components of the oral stabilization setup, including the 3D-printed holder and a disposable wooden bite support. The wooden stick was folded and inserted into the holder to create a bite interface. **(B)** The 3D-printed stabilization mount attached to the portable device. **(C)** Example of oral stabilization during testing, shown in frontal and lateral views. **(D)** Representative recording obtained using the medical-grade vHIT system. **(E)** Representative recording obtained using the portable vHIT system. Panels **(D,E)** are illustrative examples. Compared to medical-grade recordings, recordings made with the portable vHIT show blurred waveforms, making it impossible to clearly identify catch-up saccades. This reflects differences in signal characteristics and sampling methods between the two systems. **(F,G)** Illustrate a representative discordant measurement pair corresponding to the variability observed in absolute gain agreement analyses. vHIT, video head impulse test; 3D, three-dimensional.

The oral stabilization mount used a fixed holder geometry without patient-specific customization. A disposable wooden ice cream stick was folded in half and inserted into the 3D-printed holder, and participants were instructed to bite the disposable interface firmly during the brief vHIT maneuver (Figures 1A–C). The design 3D data for the holder are publicly available on the developer’s website. Although this interface is noninvasive, it requires oral contact and therefore represents a practical rather than universally applicable solution.

### 2.5 Testing protocol

Participants fixated on a visual target positioned 150 cm away. Head impulses were delivered in the horizontal plane while either the EyeSeeCam goggles or the portable device was used to record eye and head movements. At least 10 head impulses were performed in each direction. Peak head velocities were maintained between 150° and 250°/s, with amplitudes between 20° and 30°. VOR gain was calculated as the ratio of eye velocity to head velocity using the instantaneous gain at 60 ms, as determined using the EyeSeeCam software. For consistency in between-device comparisons and healthy volunteer analyses, abnormal classification was defined as a horizontal canal gain of <0.78 (8, 9). Because a validated device-specific diagnostic threshold has not been established for the portable system, this value was used as a provisional reference threshold rather than a validated portable-specific cutoff.

All tests were performed by a single examiner. The order of testing was fixed, with the medical-grade device tested first, followed by the portable system. A short rest period was allowed between measurements. For patient analyses, right and left measurements were mapped to the affected and unaffected sides according to the clinically determined side of vestibular hypofunction.

### 2.6 Outcomes and definitions

The primary analysis focused on a patient-level laterality endpoint, defined as the laterality difference (unaffected minus affected VOR gain), calculated separately for the medical-grade and portable systems. This endpoint was prespecified because unilateral vestibular hypofunction is characterized by side-to-side asymmetry; this approach avoids treating the two ears as statistically independent observations and preserves the original gain scale for direct between-device comparison. Within each system, discrimination between the affected and unaffected sides was evaluated using paired comparisons of VOR gain in patients. Method comparison was performed using the between-device difference in laterality (portable minus medical-grade system). For patients, inferential analyses of the prespecified primary endpoint were conducted at the patient level. Ear-level gain values were presented as descriptive or secondary analyses, whereas healthy-volunteer classifications were reported at both the ear and subject levels because unilateral threshold-based abnormality may occur and both perspectives are clinically informative.

Analyses in healthy volunteers were framed as abnormal (false-positive) classifications. Because a validated portable-specific diagnostic threshold is not yet available, classifications were based on the provisional reference threshold of 0.78 derived from medical-grade vHIT literature (8, 10). Abnormal proportions were summarized at both the ear and subject levels; at the subject level, a participant was considered abnormal if either ear showed a gain of <0.78. Descriptive statistics for VOR gain and within-test variability metrics were also summarized for patients and healthy volunteers.

### 2.7 Statistical analysis

Continuous variables are presented as mean ± standard deviation. Within-patient comparisons between affected and unaffected sides were performed using paired t-tests. The laterality difference was defined as the unaffected minus affected VOR gain, and the between-device difference as the portable minus the medical-grade system. Mean differences are reported with two-sided 95% confidence intervals (CIs) calculated using the *t*-distribution. Noninferiority was assessed using a prespecified margin of 0.15; noninferiority was concluded when the lower bound of the 95% CI exceeded −0.15. Agreement between systems was evaluated using Bland–Altman analysis. As a secondary analysis, direct agreement in absolute VOR gain values between the portable and medical-grade systems was evaluated using scatter plots, mean between-device differences, 95% limits of agreement, and intraclass correlation coefficients [ICC(A,1)]. These analyses were performed for affected ears, unaffected ears, healthy ears, all patient ears combined, and all ears combined. As a sensitivity analysis for absolute VOR gain agreement, we fitted a linear mixed-effects model with the between-device difference in absolute VOR gain (portable minus medical-grade) as the dependent variable, mean absolute VOR gain as a fixed effect, and subject as a random intercept. This model was used to assess systematic bias while accounting for within-subject clustering of ears and to explore potential proportional bias.

In healthy controls, abnormal proportions were calculated at the ear and subject levels with Wilson’s 95% CIs. All analyses were performed using Python 3 (pandas, NumPy, and SciPy). A two-sided *p*-value <0.05 was considered statistically significant.

## 3 Results

### 3.1 Study cohort

A total of 13 patients with unilateral vestibular hypofunction and 14 healthy volunteers were included. Patients were consecutively recruited individuals who presented to the Mejiro University Ear Institute Clinic with symptoms of dizziness or vertigo and were clinically diagnosed with unilateral vestibular hypofunction. The mean age of the patients was 66.5 ± 11.8 years, and 4 of 13 (30.8%) were female. The affected side was right in nine patients and left in four. Clinical characteristics are summarized in Table 1. The patient cohort was clinically heterogeneous and included vestibular neuritis, Ramsay Hunt syndrome, postoperative acoustic neuroma, idiopathic sudden sensorineural hearing loss with vertigo, and unilateral vestibular hypofunction of unknown cause. Symptom duration was recorded categorically, and spontaneous nystagmus findings are shown in Table 1. Healthy volunteers were independently recruited, were not age-matched to the patient group, and had no history of vestibular or neurological disease. Their mean age was 30.4 ± 13.3 years, and 7 of 14 (50.0%) were female. All participants underwent testing with both systems during the same session.

### 3.2 Descriptive statistics of VOR gain and within-test variability

Descriptive statistics are summarized in Table 2. In patients, the mean VOR gain on the affected side was 0.422 ± 0.150 for the medical-grade device and 0.452 ± 0.180 for the portable system, whereas the corresponding mean gains on the unaffected side were 0.940 ± 0.167 and 0.905 ± 0.143, respectively. In healthy volunteers, the mean VOR gain across all ears was 1.075 ± 0.102 for the medical-grade device and 1.017 ± 0.195 for the portable system.

**Table 2:** Descriptive statistics of vHIT gain in patients and healthy volunteers.

Group	*N*	Side/ear	Medical-grade device (VOR gain)	Portable system (VOR gain)
Patients	13	Affected	0.422 ± 0.150	0.452 ± 0.180
		Unaffected	0.940 ± 0.167	0.905 ± 0.143
Healthy volunteers	14	Right	1.061 ± 0.080	0.907 ± 0.201
		Left	1.089 ± 0.122	1.126 ± 0.112
	28 ears	All ears	1.075 ± 0.102	1.017 ± 0.195

vHIT, video head impulse test; VOR, vestibulo-ocular reflex. Values are presented as mean ± standard deviation. In patients, values represent affected versus unaffected sides. In healthy volunteers, values represent right versus left sides, with all ears combined.

### 3.3 Direct comparison of absolute VOR gain values between devices

**Figure 2:**
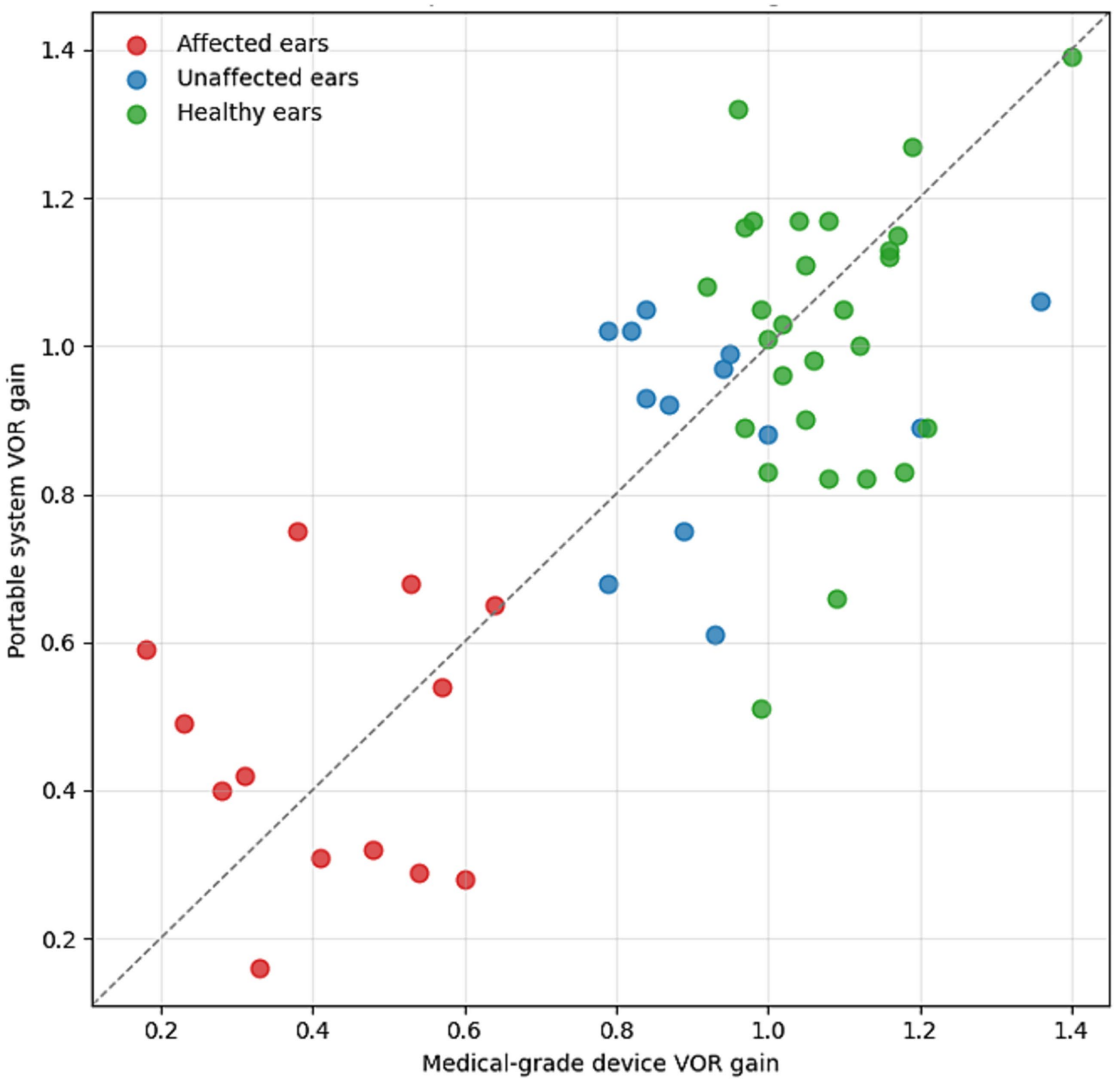
Direct comparison of absolute VOR gain values between the portable and medical-grade systems. Scatter plots show absolute VOR gain values obtained with the portable system versus the medical-grade device. Points are stratified as affected ears, unaffected ears, and healthy ears. The dashed diagonal line indicates identity. VOR, vestibulo-ocular reflex; vHIT, video head impulse test.

Scatter plots of absolute VOR gain values obtained with the portable and medical-grade systems are shown in Figure 2. Representative waveform examples from a typical case and a discordant measurement pair are shown in Figure 1D–G. Panels D and E illustrate the typical waveform characteristics of the medical-grade and portable systems, whereas panels F and G illustrate the type of individual-level discrepancy underlying the wide limits of agreement observed for absolute VOR gain. When all ears were analyzed together, absolute gain values showed moderate-to-good between-device agreement (ICC(A,1) = 0.757), with a mean difference of −0.031 (portable minus medical-grade). When patient ears were analyzed together, agreement was also moderate (ICC(A,1) = 0.745), with a mean difference of −0.002.

**Figure 3:**
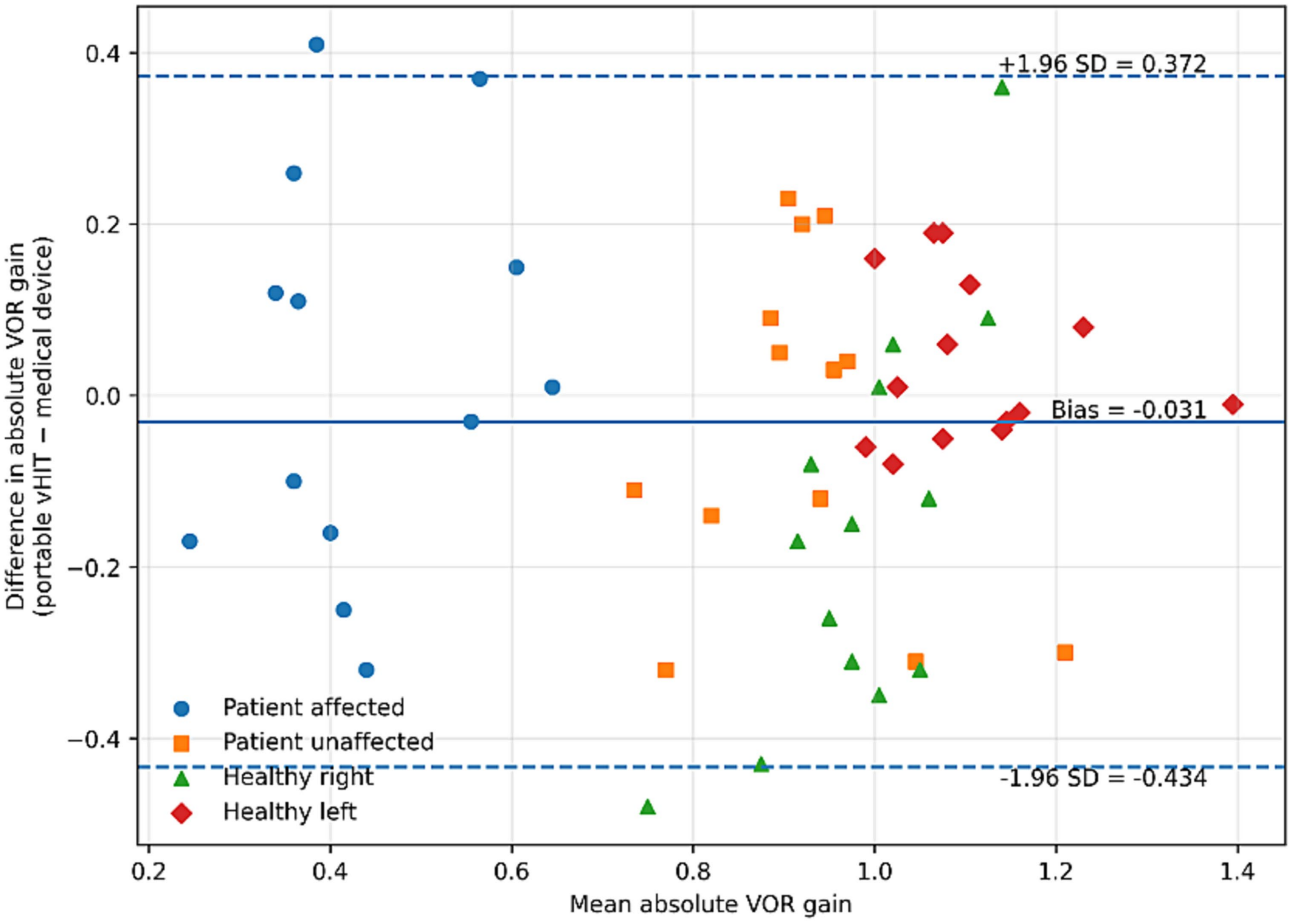
Bland–Altman plot for absolute VOR gain across all ears. The y-axis shows the difference between devices (portable minus medical-grade), and the x-axis shows the mean of the two measurements. The solid line indicates the mean bias, and the dashed lines indicate the 95% limits of agreement. Points represent affected ears, unaffected ears, healthy right ears, and healthy left ears. VOR, vestibulo-ocular reflex; vHIT, video head impulse test.

By contrast, within narrower subgroups defined by affected ears, unaffected ears, or healthy ears, ICC values were lower (0.031, 0.200, and 0.177, respectively), despite relatively small mean differences (+0.031, −0.035, and −0.058, respectively) (Table 3). These findings indicate that absolute gain values were associated across systems overall, but subgroup-level agreement was limited within narrower gain ranges, where restricted between-subject variability may reduce ICC values. A Bland–Altman plot for absolute VOR gain across all ears is shown in Figure 3. The mean between-device difference was −0.031, with 95% limits of agreement from −0.434 to 0.372, indicating small average bias but substantial individual-level variability. In a sensitivity analysis using a linear mixed-effects model with subject as a random intercept, the intercept was −0.021 (95% CI, −0.191 to 0.150; *p* = 0.811), indicating no significant overall systematic bias after accounting for within-subject clustering. The coefficient for mean absolute gain was −0.012 (95% CI, −0.193 to 0.169; *p* = 0.900), providing no evidence of proportional bias. Taken together, these findings indicate that absolute gain values showed limited average bias between systems, but agreement at the individual-ear level remained variable.

**Table 3:** Secondary analysis of direct agreement in absolute VOR gain values between the portable and medical-grade systems.

Group	*n*	Medical-grade device (mean ± SD)	Portable system (mean ± SD)	Mean difference (portable − medical)	95% LoA	ICC (A,1)
Affected ears	13	0.422 ± 0.150	0.452 ± 0.180	+0.031	−0.422 to 0.483	0.031
Unaffected ears	13	0.940 ± 0.167	0.905 ± 0.143	−0.035	−0.422 to 0.353	0.200
Healthy ears	28	1.075 ± 0.102	1.017 ± 0.195	−0.058	−0.447 to 0.331	0.177
Patients, all ears	26	0.681 ± 0.307	0.679 ± 0.281	−0.002	−0.420 to 0.416	0.745
All ears	54	0.885 ± 0.298	0.854 ± 0.293	−0.031	−0.434 to 0.372	0.757

ICC, intraclass correlation coefficient; LoA, limits of agreement; VOR, vestibulo-ocular reflex. Mean difference was defined as the portable system minus the medical-grade device. ICC(A,1) represents a two-way random-effects, absolute-agreement, single-measure intraclass correlation coefficient.

### 3.4 Discrimination between affected and unaffected sides within patients

**Figure 4:**
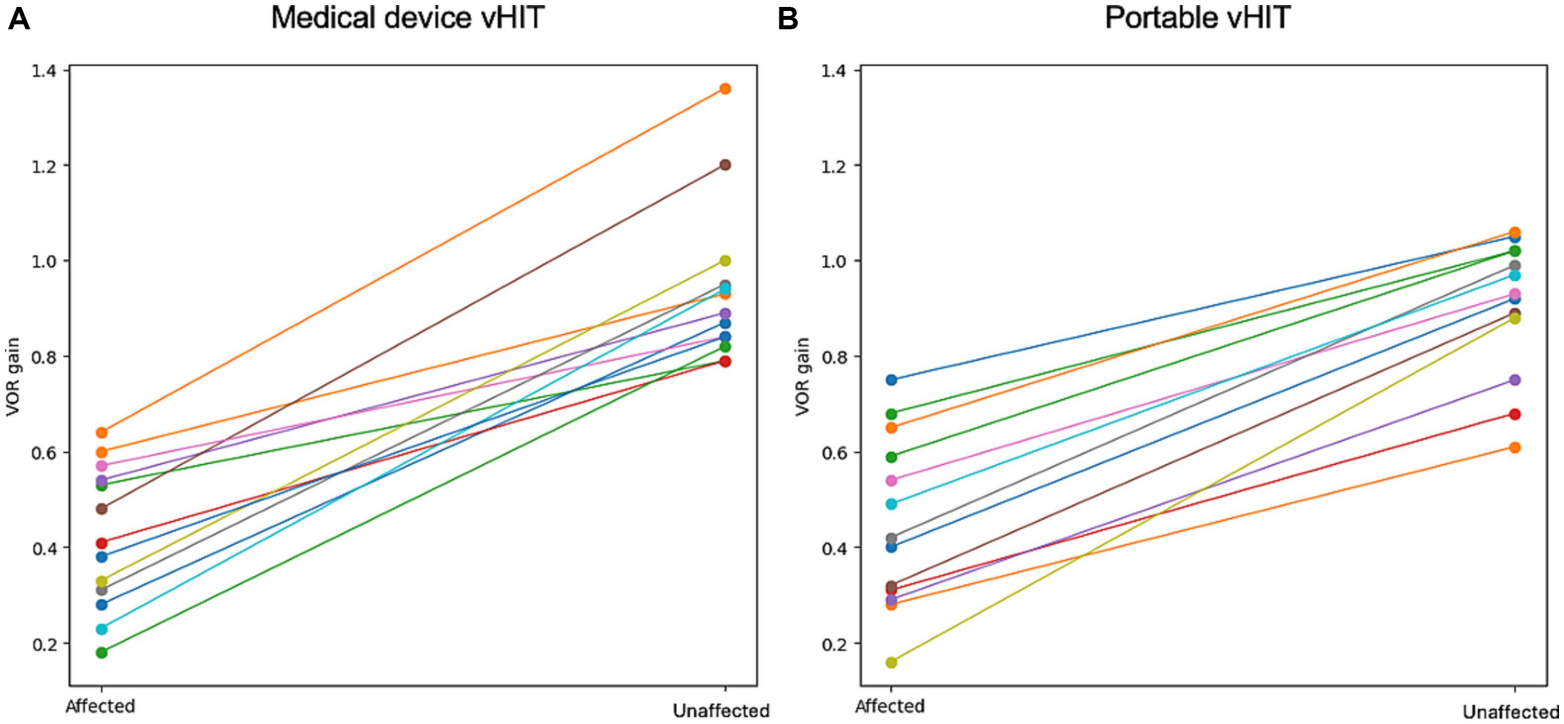
Paired comparison of VOR gain between affected and unaffected sides in patients. **(A)** Medical-grade device vHIT system. **(B)** Portable vHIT system. Each line represents an individual patient (*n* = 13), illustrating within-patient differences in VOR gain between affected and unaffected sides. Both systems demonstrate consistently higher VOR gain on the unaffected side. VOR, vestibulo-ocular reflex; vHIT, video head impulse test.

Within patients, both systems showed higher VOR gains on the unaffected side than on the affected side (Figure 4). The mean laterality difference was 0.518 ± 0.180 for the medical-grade device and 0.453 ± 0.118 for the portable vHIT system. Both within-patient comparisons were statistically significant (paired *t*-test: medical-grade device, *p* = 2.44 × 10^−7^; portable system, *p* = 1.02 × 10^−8^) (Table 4). These findings indicate that the portable system preserved the expected side-dependent asymmetry characteristic of unilateral vestibular hypofunction.

**Table 4:** Key outcomes: discriminability, noninferiority and agreement, and false-positive classification.

Domain	Outcome/metric	Medical-grade device	Portable system
Patients (*n* = 13)	Affected-side gain	0.422 ± 0.150	0.452 ± 0.180
	Unaffected-side gain	0.940 ± 0.167	0.905 ± 0.143
	Laterality difference	0.518 ± 0.180	0.453 ± 0.118
	95% CI	0.409–0.627	0.381–0.525
	Paired *t*-test *p*-value	2.44 × 10^−7^	1.02 × 10^−8^
Primary endpoint	Between-device difference	–	−0.065 ± 0.139
	95% CI	–	−0.149–0.018
	Noninferiority	–	Noninferior
	Bland–Altman bias	–	−0.065
	95% limits of agreement	–	−0.337–0.207
Healthy volunteers (*n* = 14; 28 ears)	Abnormal ears, n/N (%)	0/28 (0.0%)	2/28 (7.1%)
	95% CI	0.0–12.1%	2.0–22.6%
	Abnormal subjects, n/N (%)	0/14 (0.0%)	2/14 (14.3%)
	95% CI	0.0–21.5%	4.0–39.9%
	All-ears gain	1.075 ± 0.102	1.017 ± 0.195
	5th–95th percentile	0.964–1.203	0.716–1.303

CI, confidence interval; vHIT, video head impulse test; VOR, vestibulo-ocular reflex. Values are presented as mean ± standard deviation unless otherwise indicated. The laterality difference was defined as the unaffected minus affected VOR gain. The between-device difference was defined as the portable system minus the medical-grade device. Noninferiority was concluded when the lower bound of the 95% CI exceeded −0.15. Healthy volunteer analyses were summarized at both the ear and subject levels; at the subject level, a participant was classified as abnormal if either ear showed a gain of <0.78.

### 3.5 Noninferiority and agreement for the primary endpoint

**Figure 5:**
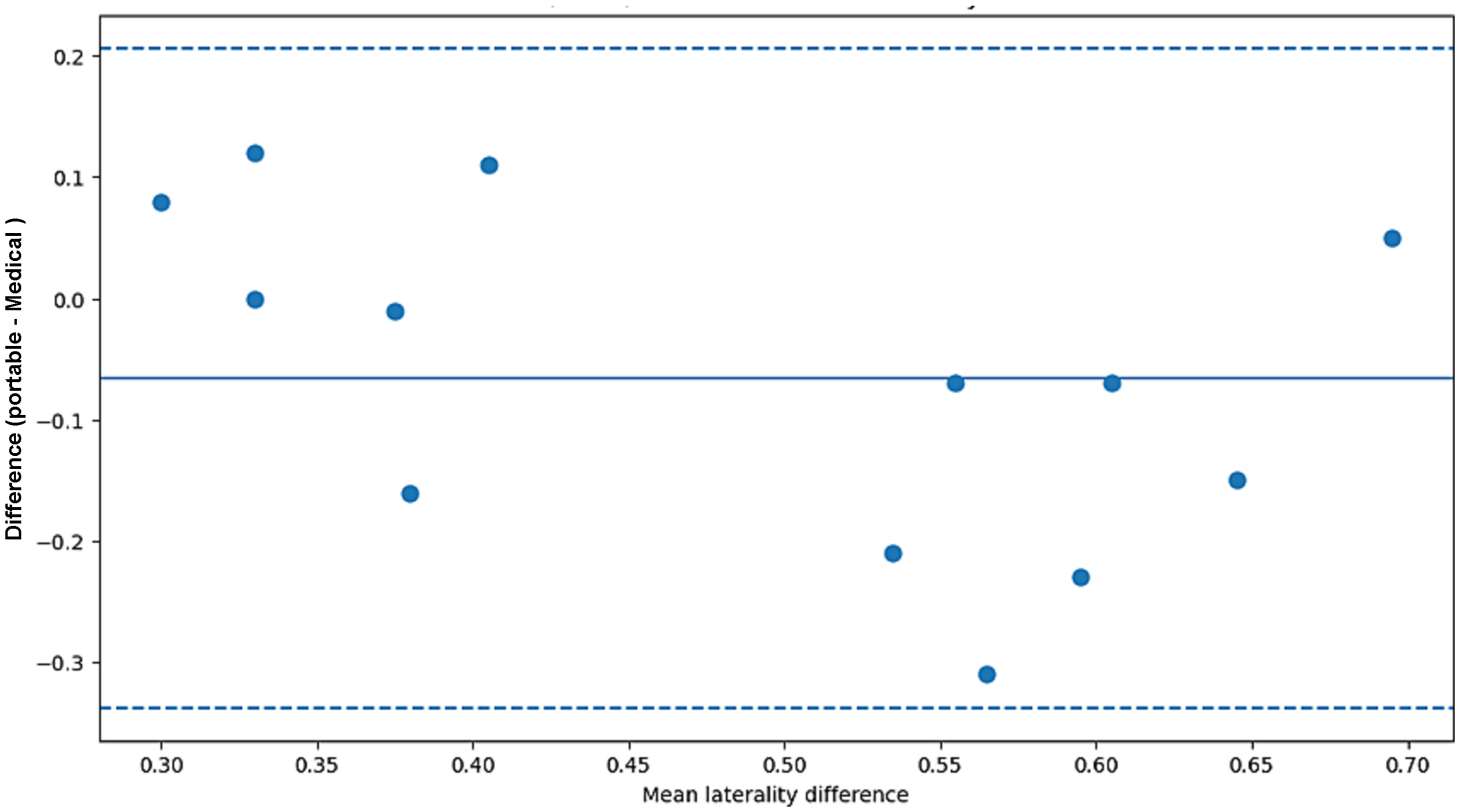
Bland–Altman plot for laterality difference in patients. The y-axis shows the difference between devices (portable minus medical-grade vHIT), and the x-axis shows the mean of the two measurements. The solid line represents the mean bias, and the dashed lines represent the 95% limits of agreement. vHIT, video head impulse test.

For the primary endpoint, the between-device difference in laterality was −0.065 ± 0.139, with a two-sided 95% CI of −0.149 to 0.018 (Table 4). Using a prespecified noninferiority margin of 0.15, the lower bound of the CI exceeded −0.15, supporting noninferiority of the portable system for the laterality difference endpoint. Bland–Altman analysis demonstrated a mean bias of −0.065, indicating that the portable system yielded slightly smaller laterality differences on average (Figure 5; Table 4). The 95% limits of agreement ranged from −0.337 to 0.207.

### 3.6 False-positive classification and reference distribution in healthy volunteers

In healthy volunteers, abnormal classification based on a provisional gain threshold of <0.78 occurred in 0 of 28 ears and 0 of 14 subjects with the medical-grade device, and in 2 of 28 ears (7.1%), corresponding to 2 of 14 subjects (14.3%), with the portable system (Table 4). One abnormal classification occurred in the right ear and one in the left ear, in two different participants.

## 4 Discussion

### 4.1 Principal findings

The aim of developing this portable vHIT system is not to replace medical-grade devices but to complement existing technologies by expanding access to quantitative vestibular assessment in settings where conventional systems are not readily available. Medical-grade vHIT systems remain essential for comprehensive vestibular evaluation, detailed waveform interpretation, and clinical decision-making at the individual patient level. This study provides quantitative evidence that a portable vHIT system using an iPod touch and an oral stabilization 3D–printed mount can detect clinically meaningful laterality in patients with unilateral vestibular hypofunction. In within-patient comparisons, both the medical-grade device and the portable system showed significantly lower VOR gains on the affected side than on the unaffected side, indicating that the portable system preserves the side-dependent asymmetry characteristic of unilateral vestibular hypofunction. Using a patient-level laterality endpoint, the portable system met prespecified noninferiority criteria relative to the medical-grade reference. The mean between-device difference was small, and Bland–Altman analysis demonstrated a slight negative bias. These findings suggest that, at the group level, the portable system can provide laterality information comparable to that of the medical-grade device, although individual-level variability remains clinically relevant.

### 4.2 Relationship to prior smartphone vHIT work

A prototype smartphone-based vHIT system previously demonstrated technical feasibility and representative waveform recording using goggle-based fixation (4, 7). That early work also highlighted the need for larger validation studies and raised concerns regarding fixation stability and slippage. The present study extends this line of research in two ways. First, it introduces an oral stabilization approach using a 3D-printed mount to improve coupling stability during head impulses. Second, it provides a quantitative comparison with a medical-grade reference system using a clinically interpretable patient-level endpoint and formal agreement analysis. In this respect, the present study advances beyond feasibility toward early-stage clinical validation. This implementation also differs practically from the earlier goggle-based approach in that the device is stabilized through a noninvasive oral interface using a disposable wooden bite support within a fixed 3D-printed mount geometry. This simple configuration may help reduce relative motion between the device and the head during horizontal impulses; however, slippage was not directly quantified in the present study.

### 4.3 Interpretation of the laterality endpoint

The laterality difference was selected as the primary endpoint because unilateral vestibular hypofunction is fundamentally characterized by asymmetry between the two sides (1). This endpoint summarizes asymmetry into a single value per patient, avoids treating the two ears as statistically independent observations, and preserves the patient as the unit of analysis. In addition, the laterality difference may reduce the influence of device-dependent differences in absolute gain values between systems compared with evaluating absolute gain values alone (10, 11). Absolute VOR gain values remain clinically important for individual interpretation; however, cross-device differences in hardware configuration, camera sampling rate, motion sensors, and signal-processing algorithms may introduce systematic differences in absolute gain values even when the same subjects are tested. In this context, an asymmetry-based endpoint may provide a more robust basis for cross-device method comparison.

In secondary analyses of absolute gain, Bland–Altman analysis showed small average bias but wide limits of agreement, and mixed-effects sensitivity analysis provided no evidence of marked systematic or proportional bias after accounting for within-subject clustering. However, these findings do not establish interchangeability of absolute VOR gain values between systems at the individual-ear level. Rather, they indicate that disagreement in absolute gain was characterized mainly by substantial individual-level variability rather than by a consistent average offset or proportional trend. Inspection of the Bland–Altman plot and scatter distributions indicates that a subset of measurement pairs showed relatively large between-device discrepancies. These discordant cases are likely to reflect a combination of factors, including subtle instability of device–head coupling, differences in tracking robustness during high-velocity impulses, and variability in impulse selection and artifact rejection between systems. Importantly, these discrepancies did not follow a consistent directional or proportional pattern, as supported by the mixed-effects analysis, suggesting that they represent sporadic measurement variability rather than systematic bias. Compared with an asymmetry ratio, laterality difference preserves the original gain scale and avoids instability when denominator values are small, while still capturing the side-to-side asymmetry that characterizes unilateral vestibular hypofunction (12).

### 4.4 Clinical implications and false-positive classification in healthy volunteers

A key requirement for broader adoption of portable vestibular tests is maintaining a low false-positive rate in healthy individuals (10, 13, 14). In the present study, two healthy ears were classified as abnormal using the portable system, whereas none were classified as abnormal using the medical-grade device. At the subject level, this corresponded to 2 of 14 healthy volunteers (14.3%), with one right ear and one left ear affected in two different individuals. Notably, these abnormal classifications occurred near the lower bound of the normal gain distribution, suggesting that borderline findings should prompt retesting rather than immediate diagnostic conclusions. In this study, a gain threshold of 0.78 was used for classification based on medical-grade vHIT literature; however, a validated device-specific threshold has not been established for the portable system. Accordingly, these findings should be interpreted as provisional threshold-based classifications rather than definitive evidence of false positivity. Differences in hardware configuration and gain-calculation algorithms across devices may also contribute to variation in absolute gain values, supporting cautious interpretation when applying fixed thresholds across systems. This does not diminish the potential utility of the portable platform but underscores the need for operational safeguards, including retesting of borderline results, standardized acquisition protocols, and application-level quality control. Although the oral stabilization mount is intended to improve device–head coupling, variability related to tracking, blinking, and impulse selection may still influence gain estimates, particularly in individuals with normal vestibular function (10, 11, 14). For real-world implementation, portable vHIT should therefore be accompanied by clearly defined quality criteria and protocols for repeated measurements. These findings also support cautious interpretation of absolute gain values near diagnostic thresholds, particularly when portable systems are used outside specialist settings.

### 4.5 Method comparison and noninferiority considerations

The noninferiority results should be interpreted alongside the agreement analysis. The confidence interval for the mean between-device difference met the prespecified noninferiority criterion, indicating that the laterality difference obtained with the portable system was not meaningfully worse than that obtained with the medical-grade device within the defined margin. However, the Bland–Altman limits of agreement demonstrate a moderate spread in individual-level differences. Clinically, this suggests that the portable system may serve as an accessible quantitative tool for laterality assessment, while measurements near decision thresholds may require repeated testing or confirmation with a conventional device. By contrast, the additional agreement analyses for absolute VOR gain indicate that, although average between-device bias was limited, individual-ear agreement remained insufficiently narrow to support direct interchangeability of absolute gain values between systems. Accordingly, the present findings support the portable system primarily as a method-comparable tool for laterality assessment at the group or screening level, rather than as a direct substitute for medical-grade vHIT in individual diagnostic decision-making based solely on absolute gain thresholds.

### 4.6 Limitations

This study has several limitations. First, the sample size was modest, and estimates of agreement and false-positive classification may differ in larger or more heterogeneous populations. Second, whereas reduced VOR gain in medical-grade vHIT is typically accompanied by clearly identifiable catch-up saccades, portable vHIT recordings in this study often appeared as less clearly delineated waveforms rather than discrete saccades. This likely reflects differences in hardware configuration, sampling characteristics, and motion stability, and suggests that waveform-level interpretation should be performed with caution when using portable systems. Accordingly, the portable system is better suited for laterality-based or quantitative screening endpoints than for detailed analysis of saccade morphology. Third, all tests were performed by a single examiner, and inter-operator variability was not assessed. Fourth, the analysis was limited to horizontal vHIT and did not address vertical canal function. Fifth, although a noninferiority margin of 0.15 was used, further work is needed to determine how such margins relate to clinical decision-making and diagnostic classification. Sixth, patient-independent benchtop validation of eye- and head-velocity accuracy was not performed. Therefore, the present findings should be interpreted as a clinical method-comparison study rather than a comprehensive engineering validation. Potential effects of camera sampling, rolling shutter, synchronization error, and motion artifacts require further evaluation under controlled conditions. Seventh, the present implementation relied on a specific hardware platform (iPod touch, 7th generation), which has since been discontinued. These results should therefore be considered platform-specific and not directly generalizable to other devices without separate validation.

Finally, false-positive classification in healthy volunteers was assessed at both the ear and subject levels; however, these estimates should be interpreted cautiously given the small sample size. In addition, the gain threshold of 0.78 was derived from medical-grade vHIT literature and has not been validated for the portable system. Device-specific reference ranges and diagnostic cutoffs should therefore be established in larger future studies. Although the oral stabilization mount is noninvasive and uses a disposable bite interface, its practicality may vary depending on user tolerance, dentition, and oral anatomy.

### 4.7 Future directions

From a broader perspective, the portable vHIT system is intended to serve as an access-enhancing tool rather than a competing diagnostic platform. It may have roles in screening, follow-up assessment, and research applications in which full-scale systems are impractical. Future studies should include larger, multicenter cohorts and evaluate repeatability, inter-operator variability, and performance across diverse clinical environments. Additional work is needed to define operational quality-control criteria within the application, including automated detection and handling of tracking loss, blinking, and suboptimal impulses. Extending validation to vertical canal testing and a broader spectrum of vestibular disorders will also be important. Further work should include benchtop validation under controlled conditions, as well as hardware-specific verification on currently available devices, to determine how broadly this approach can be generalized beyond the present iPod touch–based implementation.

## 5 Conclusion

A portable vHIT system using an iPod touch and an oral stabilization 3D-printed mount detected clear laterality in unilateral vestibular hypofunction and met prespecified noninferiority criteria relative to a medical-grade vHIT system for a patient-level laterality endpoint. Agreement analysis demonstrated a small mean bias, alongside moderate variability at the individual level. These findings support the feasibility of this portable approach and justify further evaluation of its clinical validity in larger populations. Overall, the results support its role as an access-enhancing and quantitatively informative platform for laterality assessment, while indicating that absolute VOR gain agreement at the individual-ear level remains limited.

## Data Availability

The raw data supporting the conclusions of this article will be made available by the authors, without undue reservation.
